# Multi-species transcriptome analyses for the regulation of crocins biosynthesis in Crocus

**DOI:** 10.1186/s12864-019-5666-5

**Published:** 2019-04-27

**Authors:** Oussama Ahrazem, Javier Argandoña, Alessia Fiore, Andrea Rujas, Ángela Rubio-Moraga, Raquel Castillo, Lourdes Gómez-Gómez

**Affiliations:** 1 Instituto Botánico, Departamento de Ciencia y Tecnología Agroforestal y Genética, Universidad de Castilla-La Mancha, Campus Universitario s/n, 02071 Albacete, Spain; 2 Italian National Agency for New Technologies, Energy, and Sustainable Development, Casaccia Research Centre, 00123 Rome, Italy; 3 VITAB Laboratorios. Polígono Industrial Garysol C/ Pino, parcela 53, 02110 La Gineta, Albacete, Spain

**Keywords:** Apocarotenoids, Carotenoids, Carotenoid cleavage dioxygenases, Crocins, Stigmas, Transcription factors

## Abstract

**Background:**

Crocins are soluble apocarotenoids that mainly accumulate in the stigma tissue of *Crocus sativus* and provide the characteristic red color to saffron spice, in addition to being responsible for many of the medicinal properties of saffron. Crocin biosynthesis and accumulation in saffron is developmentally controlled, and the concentration of crocins increases as the stigma develops. Until now, little has been known about the molecular mechanisms governing crocin biosynthesis and accumulation. This study aimed to identify the first set of gene regulatory processes implicated in apocarotenoid biosynthesis and accumulation.

**Results:**

A large-scale crocin-mediated RNA-seq analysis was performed on saffron and two other Crocus species at two early developmental stages coincident with the initiation of crocin biosynthesis and accumulation. Pairwise comparison of unigene abundance among the samples identified potential regulatory transcription factors (TFs) involved in crocin biosynthesis and accumulation. We found a total of 131 (up- and downregulated) TFs representing a broad range of TF families in the analyzed transcriptomes; by comparison with the transcriptomes from the same developmental stages from other Crocus species, a total of 11 TF were selected as candidate regulators controlling crocin biosynthesis and accumulation.

**Conclusions:**

Our study generated gene expression profiles of stigmas at two key developmental stages for apocarotenoid accumulation in three different Crocus species. Differential gene expression analyses allowed the identification of transcription factors that provide evidence of environmental and developmental control of the apocarotenoid biosynthetic pathway at the molecular level.

**Electronic supplementary material:**

The online version of this article (10.1186/s12864-019-5666-5) contains supplementary material, which is available to authorized users.

## Background

Carotenoids are isoprenoid molecules that typically contain 40 carbons in their backbones and a number of conjugated double bonds that allow carotenoids to absorb light in the visible spectra, yielding yellow, orange, and red colors. Carotenoids are involved in a wide range of processes in plants, including growth and development, responses to environmental stimuli, photosynthesis (as accessory pigments) and attracting pollinators and seed dispersers; but also in animals, carotenoids control a wide range of physiological processes [1]. Carotenoids serve as precursors of apocarotenoids, which act as signaling molecules for plant development and to mediate responses to environmental cues [2]. Among apocarotenoids, crocins, glucosyl esters of crocetin, are water-soluble metabolites that accumulate at high levels in the stigma of *Crocus sativus*, where they function as visual signals for pollinators, due to the bright red color they provide to this tissue [3]. Crocins are also responsible for the red color of saffron spice, also known as red-gold due to the high price that it reaches in the market (5000 €/kg, www.doazafrandelamancha.com). In addition to the contribution of crocins to the color of saffron spice, these apocarotenoids have been shown to be effective in the management of neurodegenerative and psychiatric disorders [4, 5], coronary artery diseases, bronchitis, asthma, diabetes, and cancer [6]. Therefore, crocins have the potential to regulate a broad spectrum of critical cellular functions, thus influencing human health.

Earlier, it was proposed that crocins were derived from the carotenoid zeaxanthin by a 7,8;7′,8′ cleavage [7]. More recently, the enzyme responsible for this cleavage has been elucidated [8, 9], and it has been shown to produce crocetin, which is the substrate of glucosyltransferase enzymes that catalyze the production of crocins [10]. The biosynthesis and accumulation of crocins in the stigma of saffron and in flowers of other Crocus species showed an increase parallel to the expression of precursor carotenogenic and apocarotenogenic genes [11–15], which represent a chromoplast-specific carotenoid pathway for crocin biosynthesis in Crocus [3]. In plants, different strategies to control carotenoid biosynthesis and accumulation have been reported [16–18], and among them, transcriptional regulation of carotenogenic gene expression has been shown to be the major mechanism by which the biosynthesis and accumulation of specific carotenoids are regulated. However, more recently, a mechanism for posttranscriptional regulation came into the spotlight [19]. Further, epigenetic regulation of genes involved in carotenoid synthesis and degradation, including histone- and/or DNA-methylation, and RNA silencing at the posttranscriptional level affect carotenoids in plants.

A drastic change in gene expression is usually driven by transcription factors, which are master-control proteins regulating activation/suppression of gene expression through binding to specific regulatory sequences of target genes. However, the mechanisms responsible for these transcriptional controls in different plant species and tissues remain poorly understood. In addition to the role played by developmental cues, crocin biosynthesis is also affected by temperature, light and circadian rhythms [20].

Given that there is currently no reference genome available for any Crocus species, transcriptomes are key to facilitating research on secondary metabolite pathways. Efforts by independent saffron research groups have generated de novo transcriptome assemblies from different tissues of *Crocus sativus*, including leaves, stamens, corm, tepals, and stigmas (105,269 transcripts in leaf, corm, tepal, stamen and stigma [21]; 64,438 transcripts in flowers [22]; and 248,099 transcripts in tepals of *Crocus ancyrensis* at two developmental stages [15]). These transcriptome analyses on Crocus species [15, 21, 22] have unveiled thousands of transcription factor-coding genes, providing a foundation for investigating their involvement in apocarotenoid metabolism. However, in the specific case of saffron, data are only available from mature stigmas, thus we are lacking information on the critical stages of apocarotenoid biosynthesis [13].

A systematic comparative analysis approach for transcriptomes and crocin data is presented here to identify putative transcription factors that may affect apocarotenoid accumulation during stigma development in saffron. The pattern of accumulation of crocins and the expression of carotenoid- and apocarotenoid-related genes together with those coding for putative transcription factors has been analyzed in two key developmental stages of three Crocus species in order to clarify the mechanism influencing the biosynthesis and accumulation of these bioactive metabolites.

## Results and discussion

### Experimental Design of Transcriptome Analysis

**Fig. 1:**
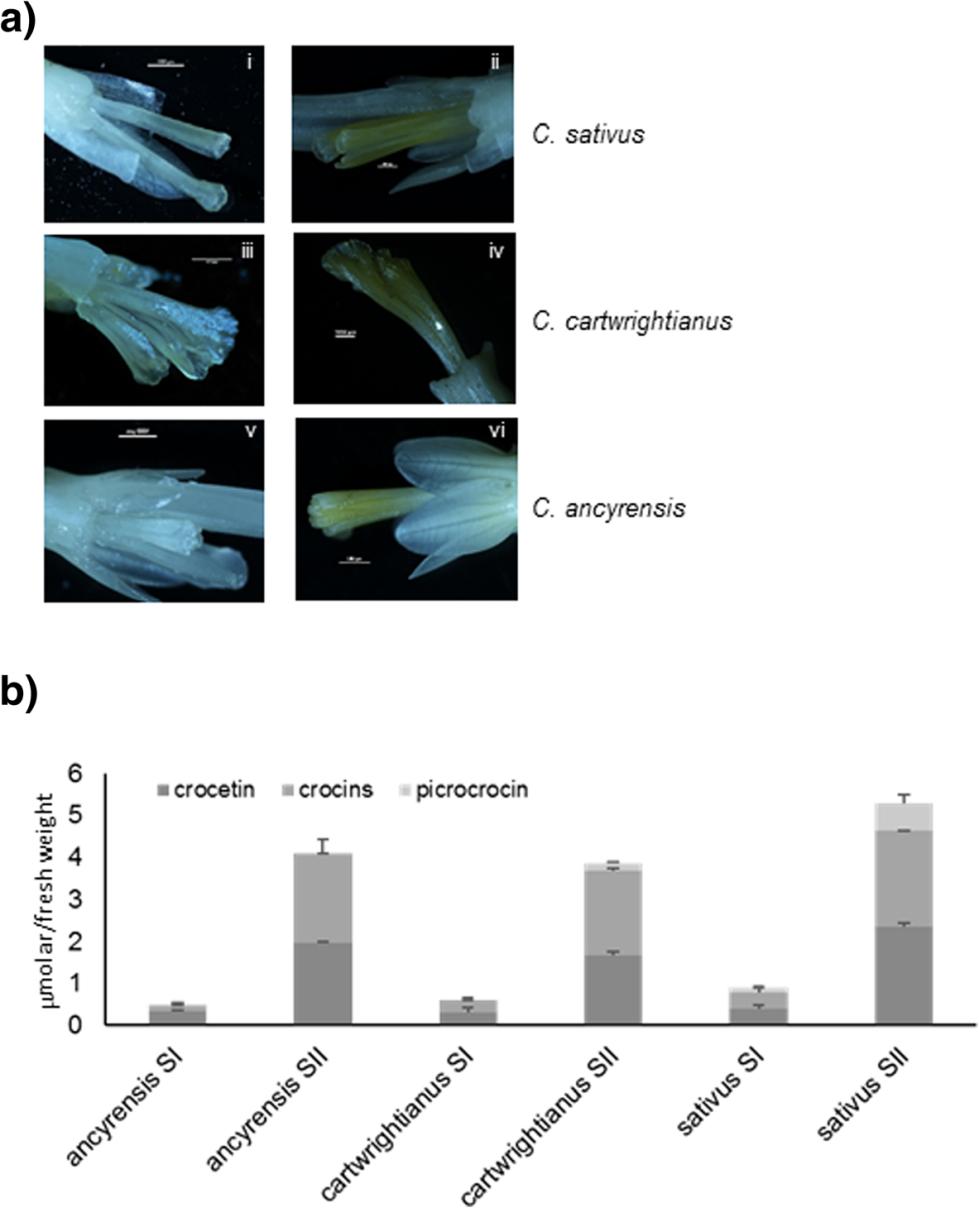
Differential accumulation of crocins and accumulation in two developmental stages of stigmas from Crocus. **a**) Stigmas in stage I and stage II from *C. sativus* (i and ii), *C. cartwrightianus* (iii and iv), and *C. ancyrensis* (v and vi). **b**) The stigmas in stage II present a distinctive yellow colouration due to the accumulation of different apocarotenoids in this stage in all the Crocus species

Three Crocus species were selected for the identification of putative TFs involved in the metabolism of crocins (Table 1). *C. sativus* shows flowers similar in size to those of *C. cartwrightianus*, however its flowers show a larger stigma. *C. ancyrensis* is characterized by smaller flowers compared with those from the Crocus blooming in autumn, and also a much smaller stigma. In terms of crocins accumulation, *C. sativus* accumulates more crocins than the other two. It is clear that ploidy in saffron is an advantage regarding crocins production. Therefore, *C. sativus* was selected as the source of saffron, and *C. cartwrightianus* was selected as a species closely related to saffron and considered to be one of the ancestors of saffron [11, 23]. Both *C. sativus* and *C. cartwrightianus* belong to section Crocus, which are species that bloom in autumn and only accumulate crocins in the stigma tissue [24]. Finally, *C. ancyrensis,* a spring flowering species that belongs to section Nudiscapus and accumulates crocins both in stigmas and tepals, was also included [14, 25]. We dissected stigmas of these three Crocus species at two developmental stages particularly focused on the transition from white to yellow stigmas because it is the beginning of crocetin biosynthesis and crocin accumulation (Fig. 1a). For each sample, we collected twenty stigmas to reduce possible biological variability as much as realistically possible. The presence of apocarotenoids was evaluated in the white (SI) and yellow (SII) stigmas of these three species by UPLC-DAD-MS analyses (Fig. 1b). For all the three species analyzed, stage SI was characterized by reduced levels of crocetin, crocins, and picrocrocin. Picrocrocin was detected in the SI stage of *C. sativus* and *C. cartwrightianus,* but not in *C. ancyrensis* SI stigmas (Fig. 1b), as previously observed [14]. Several explanations are possible; among them, the simplest could be the absence in this species of the glucosyltransferase transferring the sugar on the picrocrocin precursor (4-hydroxy-2,6,6-trimethyl-1-cyclohexene-1-carboxaldehyde). This glucosyltransferase has not yet been isolated from the autumn species, so its presence or absence cannot be determined yet in the spring crocuses. On the other hand, we cannot rule out the presence in these spring species of a glucosidase acting over picrocrocin in a very efficient way preventing its accumulation and therefore its detection. In the SII stage there was an increase in the content of crocetin and crocins in all the analyzed species, and again, picrocrocin was not detected in the stigmas of *C. ancyrensis,* as previously described [14]. In the three species, the apocarotenoid analyses revealed an increase in apocarotenoid concentration from stage I (white stigmas) to stage II (yellow stigmas).

**Table 1:** Comparison of features among the Crocus species used in this study

Species	Flowering period	Tepal color	Stigma color	Chromosome number 2n=	Distribution
*Crocus sativus*	autumn	purple	red	24	Not known as a wild plant
*Crocus cartwrightianus*	autumn	purple	red	16	Greece
*Crocus ancyrensis*	end of winter-early spring	orange	orange	10	Turkey

### Functional annotation

The assembled transcriptomes (Table 2) were used as queries for annotation by means of BLASTX searches based on sequence homologies in the National Center for Biotechnology Information (NCBI) (https://www.ncbi.nlm.nih.gov/) nonredundant protein database (nr), a public database, using Blast2GO with an E-value cut-off of 1e^− 06^. For GO analysis, annotated unigenes were divided into three main ontologies: biological process, makes reference to the biological objective of the genes or the gene products; cellular components, makes reference to the place in the cell where the gene encoding product is active; and molecular function, defined by the biochemical activity [26]. Table 3 shows the gene ontology annotation of the assembled unigenes from the 6 transcriptomes. Among the biological process terms, protein metabolism process (20–21%) was the most represented, followed by response to stimulus (12–15%) and biological regulation (11%) (Table 3). In the cellular component category, the dominant subcategory was the cell part (46–41%), followed by the organelle (14–29%) and the membrane (15–7%). Under molecular function, the term binding (42–32%) was the most represented, followed by catalytic activity (33–32%), transport activity (5%) and nucleic acid binding transcription factor activity (3%) (Table 3).

**Table 2:** Summary for RNA-Seq reads mapping and assembly of Crocus species transcriptome sequences

samples	Total raw reads	Clean reads	Q20%	GC%
sativus-SI	56,940,508	54,756,172	95.9	56.71
sativus-SII	63,508,296	61,375,566	95.56	47.79
cartwrightianus-SI	52,220,890	50,438,172	95.39	47.79
cartwrightianus-SII	53,966,626	52,375,782	95.92	47.32
ancyrensis-SI	51,968,850	50,467,394	96.1	46.42
ancyrensis-SII	53,403,894	51,852,166	96.1	46.62

**Table 3:** Gene Ontology (GO) analysis of transcriptomes associated with crocins accumulation in Crocus

Sample	Biological process	Cellular component	Molecular function	No hits
sativus-SI	19% 21% Metabolic process 13% Response to stimulus 11% Biological function	16% 41% Cell part 14% Organelle 13% Membrane	20% 42% Binding 32% Catalytic activity 3% Nucleic acid binding transcription factor activity	45%
sativus-SII	18% 21% Metabolic process 12% Response to stimulus 11% Biological function	16% 41% Cell part 14% Organelle 15% Membrane	19% 41% Binding 32% Catalytic activity 3% Nucleic acid binding transcription factor activity	48%
cartwrightianus-SI	16% 20% Metabolic process 15% Response to stimulus 11% Biological function	15% 46% Cell part 29% Organelle 7% Membrane	11% 33% Catalytic activity 32% Binding 5% Transporter activity 3% Nucleic acid binding transcription factor activity	58%
cartwrightianus-SII	16% 20% Metabolic process 14% Response to stimulus 11% Biological function	15% 46% Cell part 29% Organelle 7% Membrane	11% 32% Catalytic activity 32% Binding 5% Transporter activity 3% Nucleic acid binding transcription factor activity	59%
ancyrensis-SI	17% 20% Metabolic process 15% Response to stimulus 11% Biological function	15% 46% Cell part 29% Organelle 7% Membrane	11% 33% Catalytic activity 32% Binding 5% Transporter activity 3% Nucleic acid binding transcription factor activity	57%
ancyrensis-SII	16% 20% Metabolic process 14% Response to stimulus 11% Biological function	15% 46% Cell part 29% Organelle 7% Membrane	10% 33% Catalytic activity 32% Binding 5% Transporter activity 3% Nucleic acid binding transcription factor activity	59%

We determined the 10 most abundant transcripts present in each analyzed transcriptome by the conversion of assembled read counts into normalized digital transcript levels (Fragments Per Kilobase of exon per Million fragments mapped (FPKM) (Table 4 and Additional file 1: Figure S1). Transcript abundance varied over 6 orders of magnitude, with FPKM values ranging from 0.01 to 5269.14. Transcripts with very high transcript abundance are listed in Table 4. Among them, the translationally controlled tumor protein (TCTP) was found to be highly expressed in all the transcriptomes. TCTP belongs to a family of calcium- and tubulin-binding proteins, and it is generally regarded as a growth-regulating protein in plants [27]. A number of genes encoding for ribosomal proteins were also detected among all the transcriptomes (60S ribosomal protein L2, 60S ribosomal protein L13, and ribosomal protein S27a). Other transcripts with high abundance include histone and histone modulating enzymes, histone H2A and histone deacetylase (HDA3; HD2C) and heat shock proteins (HSP81–2, HSP81–3, HSP90–2), which probably are involved in chloroplast sorting of nuclear encoded proteins by interactions with other chaperones [28]. In the transcriptomes of *C. sativus,* several genes encoding mitochondrial proteins were found that were not present within the ten more expressed contigs of the other four transcriptomes. Among them were cytochrome C assembly protein, cytochrome c oxidase, subunit III (complex IV), cytochrome b, and ATPase, F0 complex. In the transcriptomes of *C. ancyrensis* and *C. cartwrightianus,* several lipid transfer proteins (LTP) were identified, which were also previously detected at high levels of expression in the transcriptome of *C. ancyrensis* [15] and in the stigmas of saffron [29]. LTPs are abundantly expressed in most plant tissues where they actively participate in lipid barrier deposition and cell expansion [30]. In *C. ancyrensis*, we also found contigs with identity to late embryogenesis abundant proteins (LEA), as described earlier for *C. sieberi* [15]. Most LEA proteins play an important role in abiotic stress response and stress tolerance in plants [31]. In both species, the presence of highly expressed LEA transcripts could reflect the requirement in these spring-flowering species for cold to break flower bud dormancy, as observed in other flowers’ buds [32].

### Expression of carotenogenic and apocarotenogenic genes in white and yellow stigmas

**Fig. 2:**
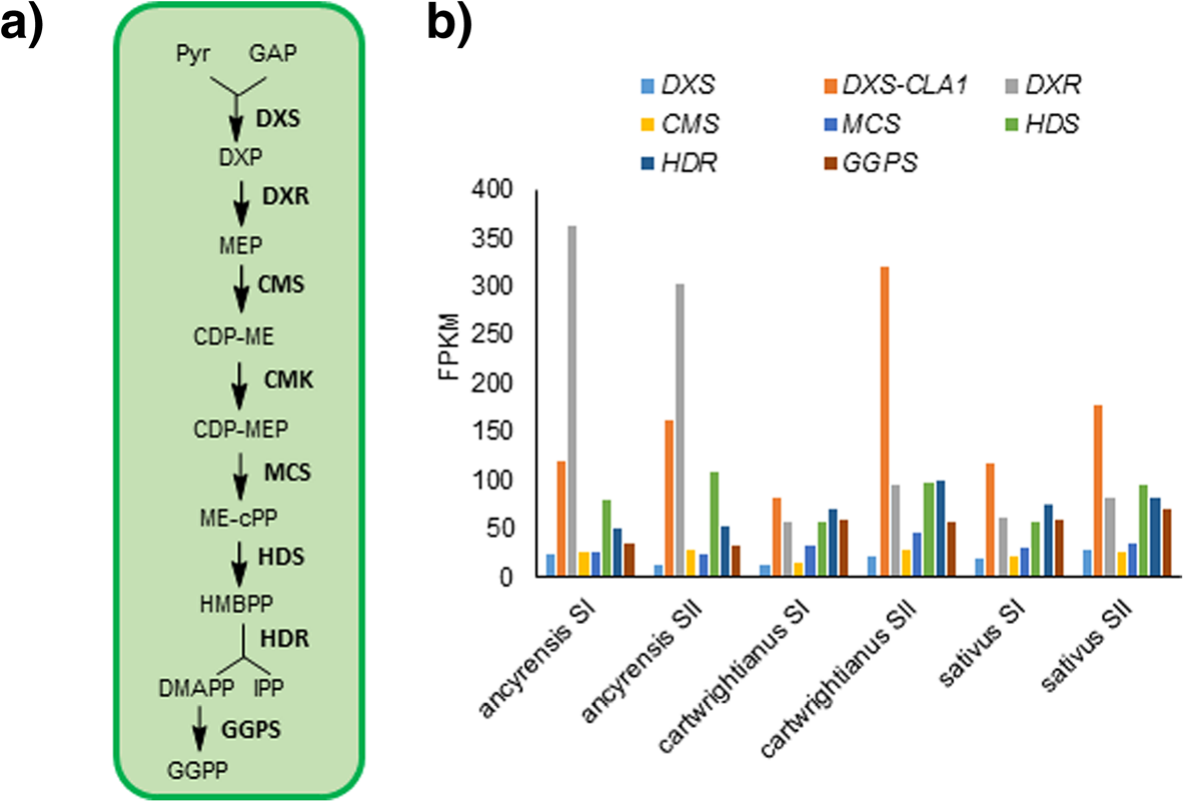
Expression levels of differentially expressed unigenes assigned to the MEP pathway. **a**) An overview of the MEP pathway. **b**) Homologues genes encoding for the different enzymes of the pathway were identified in the transcriptome assembly of stigmas at stages I and II in the three Crocus species

**Fig. 3:**
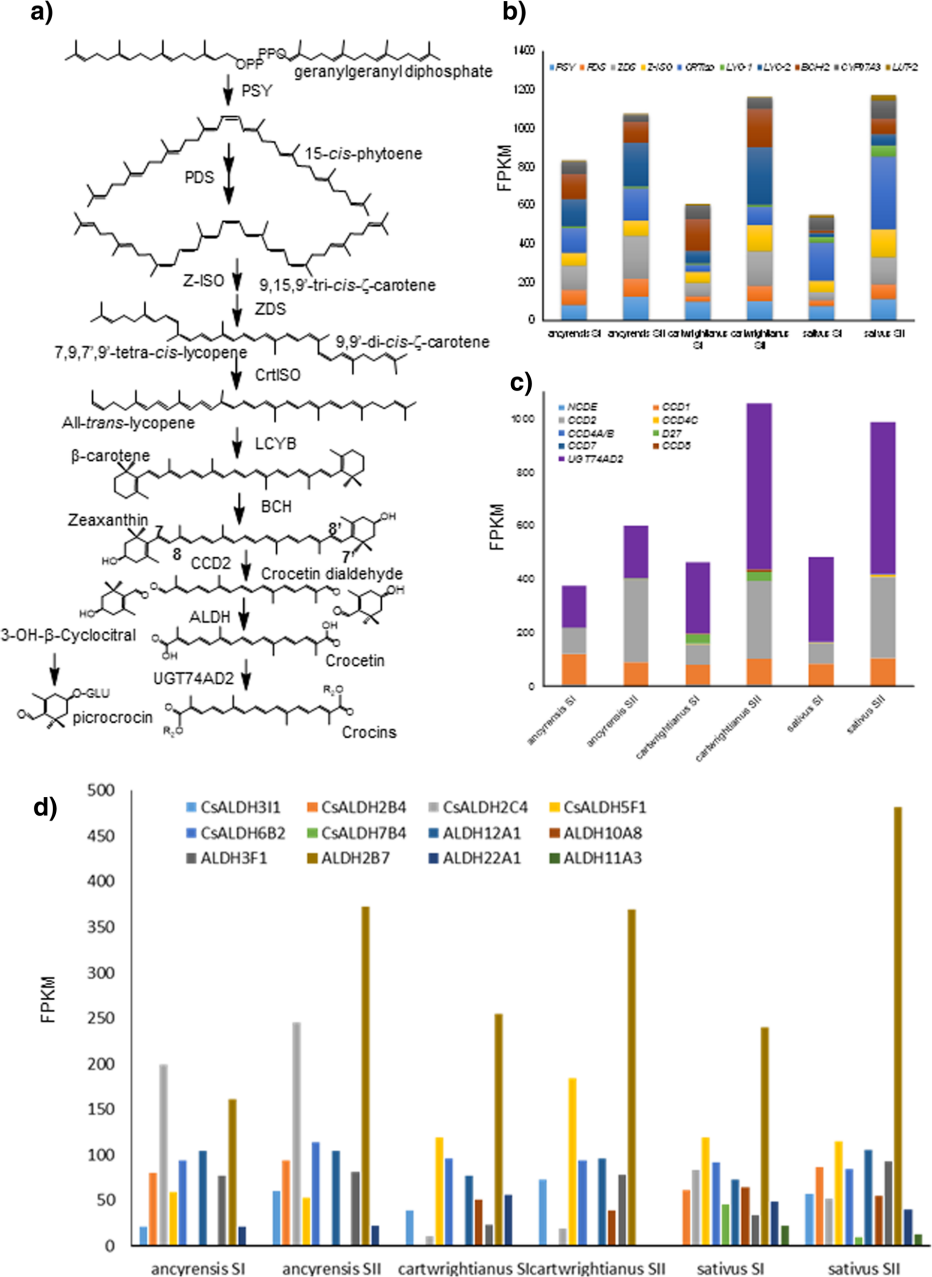
Expression levels of unigenes assigned to the carotenoid and apocarotenoid biosynthetic pathways in Crocus. **a**) An overview of the crocins biosynthesis pathway enzymes and metabolites in Crocus. Homologues genes encoding for the different enzymes were identified in the transcriptome assembly. **b**) Expression analyses of genes encoding from the enzymes of the carotenoid biosynthesis pathway identified in the transcriptomes from I and II stages of the three Crocus species. **c**) Expression analyses of homologues to carotenoid cleavage enzymes (CCD1, CCD2, CCD4, CCD7, CCD8 and NCED), to the β-carotene isomerase D27 and UGT74AD2 genes identified in the six transcriptomes. d) Expression analyses of ALDH genes homologues identified in the six transcriptomes analysed

Carotenoids are synthesized in plastids from metabolic precursors provided by methylerythritol 4-phosphate (MEP) [1]. An expression analysis of genes involved in carotenoid and apocarotenoid pathways in the three species of Crocus was performed. We started with a search for genes encoding enzymes involved in the MEP pathway. A total of eight sequences coding for putative proteins of this pathway were identified in the six transcriptomes (Fig. 2a). 1-Deoxy-D-xylulose-5-phosphate synthase (DXS) has been shown to catalyze one of the rate-limiting steps of the MEP pathway [33]. It generates 1-deoxy-D-xylulose-5-phosphate (DXP) by the condensation of pyruvate and D-glyceraldehyde 3-phosphate (Fig. 2a). DXS is typically encoded by a small gene family. High expression levels of contigs with identity to CLA1 were found in the six transcriptomes, and the expression levels increased from SI to SII (Fig. 2a). The remaining identified sequences did not show a clear repetitive pattern among the analyzed species, with the exception of hydroxymethylbutenyl diphosphate synthase (HDS), with increased expression levels from SI to SII. It has been suggested that the enzymes HDS and HDR can also contribute to the regulatory mechanisms of the MEP pathway. Several recent studies have also demonstrated that MEcPP, the substrate for HDS, is a key intermediate in the MEP pathway. This metabolite leads to a retrograde signal regulating the expression of nuclear-encoded, stress-responsive genes for plastidial proteins [34]. Carotenoid biosynthesis starts from the condensation of two geranylgeranyl diphosphate (GGPP) molecules in phytoene by phytoene synthase (PSY) (Fig. 3a) [16]. In the three species analyzed, *PSY* levels increased from stage SI to stage SII (Fig. 3b). Next, a series of desaturation and isomerization reactions catalyzed by phytoene desaturase (PDS), ζ-carotene desaturase (ZDS), ζ-carotene isomerase (Z-ISO), and carotenoid isomerase (CrtISO) lead to the biosynthesis of lycopene (Fig. 3a). All the genes encoding for these enzymes were upregulated in SII (Fig. 3b); in particular, the levels of these genes were high in SII stigmas of *C. sativus* (Fig. 3b). Cyclization of lycopene by lycopene ɛ-cyclase (LYC-E) and/or lycopene β-cyclase (LYC-B) produces α-carotene and β-carotene, respectively (Fig. 3a). Only contigs with homologies to *LYC-B* were identified in the six transcriptomes. In Crocus species, two *LCY* genes have been identified, one of them being *LCY-2*, which is chromoplast-specific [12, 14, 15]. Higher levels of expression were found for *LCY-2* in SII in all the species (Fig. 3b). Subsequent hydroxylation of α-carotene and β-carotene by two nonheme carotene hydroxylases (BCH-1 and BCH-2) and two heme hydroxylases (CYP97A (Lut-1) and CYP97C (Lut-2)) generates zeaxanthin and lutein, respectively (Fig. 3a). Similarly, to LCY-2, BCH-2 is also a chromoplast-specific enzyme [3, 11]. The expression levels of *BCH-2* increased from SI to SII. However, the levels of *Lut-1* and *Lut-2* decreased in *C. cartwrightianus* and *C. ancyrensis* from SI to SII, while in *C. sativus* the FPKM values increased from SI to SII (Fig. 3b). Further, the levels of contigs with identities to apocarotenogenic genes from saffron including *CCD1*, *CCD*2, *CCD4a/b*, *CCD4c*, *CCD7* and *CCD8* [35–37] and to β-carotene isomerase (D27) were also evaluated (Fig. 3c). The levels of contigs with identity to *CCD4a/b* and *CCD4c* were very low in all the six transcriptomes in these early developmental stages (Fig. 3c). *CCD8* was only detected in *C. cartwrightianus*, and *D27* was detected in *C. cartwrightianus* and in *C. ancyrensis*, but at very different levels (Fig. 3c). The high levels of *D27* in *C. cartwrightianus* and the low levels of *CCD8*, together with the absence of *CCD7* contigs, suggested the involvement of cis-β-carotene as a substrate for other enzymes [38]. Finally, the contigs with the highest FPKM values correspond to those encoding CCD1 and CCD2 enzymes. While *CCD1* values remain almost stable between the two developmental stages (Fig. 2c), *CCD2* levels increased more than 2.5-fold in *C. ancyrensis*, and fourfold in *C. cartwrightianus* and *C. sativus,* from SI to SII (Fig. 3c). The levels of contigs encoding UGT74AD2, the enzyme that catalyzes the glucosylation of crocetin [10], were also evaluated. In all three species, the levels increased from SI to SII at least twofold (Fig. 3c) showing a positive correlation with crocins accumulation. Further, all the contigs encoding putative aldehyde dehydrogenase (ALDH) enzymes were also analyzed (Fig. 3d). Several ALDH enzymes have been characterized in saffron previously [39–41], suggesting the promiscuity of ALDH enzymes for crocetin transformation [42]. Different FPKM values were observed for a total of 12 contigs encoding ALDHs in saffron, *C. cartwrightianus* and *C. ancyrensis*. The highest values were observed for *CsALDH2B7* (KU577906.2), which also increased its levels from SI to SII (Fig. 2d). The other *ALDHs* showed variable levels among the three species analyzed and between the two developmental stages (Fig. 3d). CsALDH3IH (KU577904) and CsALDH2B4 (KU577907), have been previously shown to catalyze the conversion of crocetin dialdehyde to crocetin in vitro [40, 41]; however, due to the reduced expression of the respective genes in SI and SII stigmas, we doubt that either of these proteins is specifically responsible for the conversion of crocetin dialdehyde to crocetin. By contrast, ALDH2B7 was highly expressed in the analyzed stigmas and showed co-expression with CCD2.

### Major transcription factor families related to apocarotenoid accumulation

In this study, 590 and 617 TFs were identified in the white (SI) and yellow (SII) transcriptomes of saffron, respectively, and those TFs belong to 102 TF families (Additional file 2: Table S1). The basic helix-loop-helix (bHLH) family was the dominant TF family in both stages, having 53 and 39 TFs in the white (SI) and yellow (SII) stages, respectively (Additional file 2: Table S1). The bHLH proteins are a superfamily of TFs found throughout eukaryotic organisms that bind to DNA as a dimer and are characterized by the presence of a 50–60 amino acid bHLH domain. They are involved in a myriad of regulatory processes, including modulation of secondary metabolism pathways, epidermal differentiation, and responses to environmental factors in plants [43, 44]. The MYB family was the second major TF family in both stages, with 29 and 36 TFs in the white and yellow stages (I and II), respectively (Additional file 2: Table S1). In addition, MYB-like TFs were also detected at relatively high levels, 21 and 15 in stages I and II, respectively (Additional file 2: Table S1). MYB represents a family of proteins that include a 52 amino acid conserved MYB DNA-binding domain and are involved in cell cycle regulation, cell proliferation, development, hormone signaling, and abiotic stress responses [45]. The next most abundant group was represented by TFs with the ZIP domain: HD-ZIP (21 in SI and 19 in SII) and bZIP (20 in SI and 22 in SII). The basic leucine (Leu) zipper (bZIP) TF family is characterized by a conserved 60–80 amino acid bZIP domain. These TFs are involved in organ and tissue differentiation, seed maturation, floral transition and initiation, vascular development and in signaling in response to abiotic/biotic stimuli [46]. The HD-ZIP proteins have an HD domain that binds the DNA and a Zip located downstream of the HD, which acts as a dimerization motif. TFs from this family have essential functions for plant development and plant responses to environmental conditions [46].

**Fig. 4:**
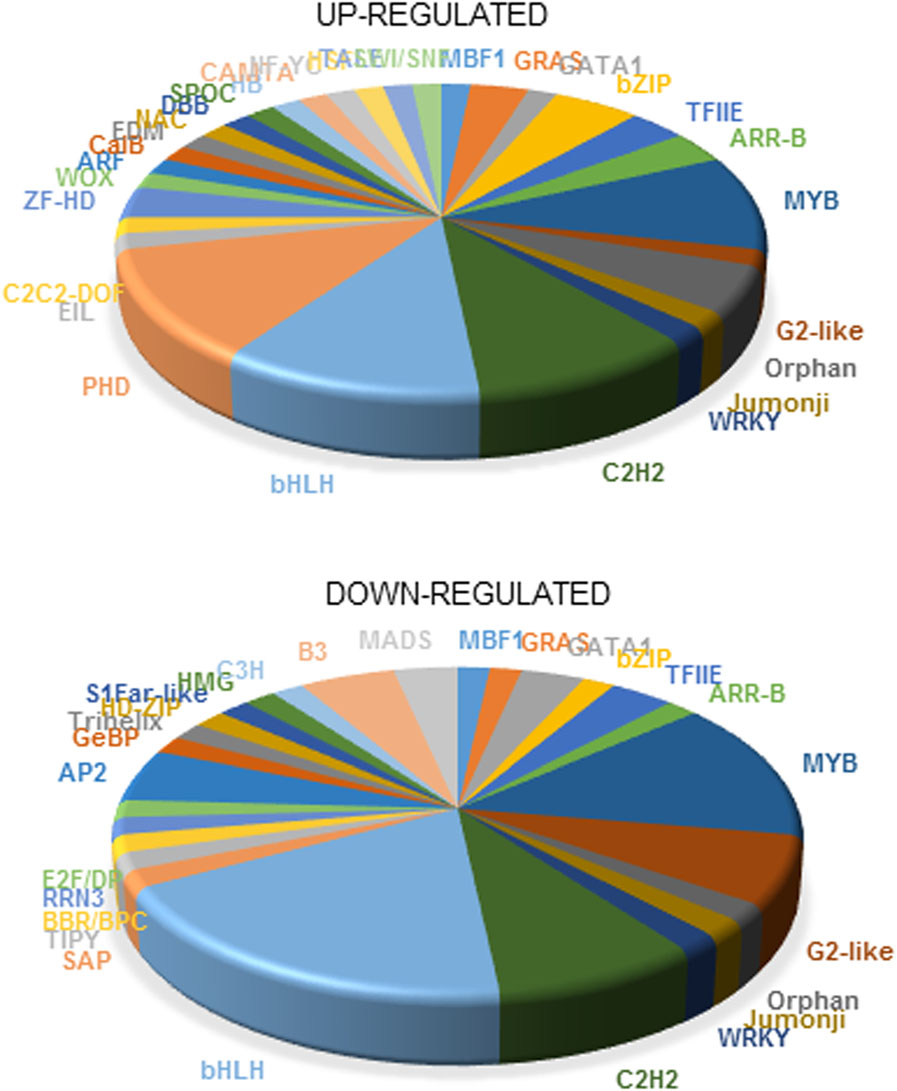
Transcription factor families identified in the differential expression analyses of the two stigmas transcriptomes of saffron. Transcription factors (TFs) were identified by sequence comparison of saffron transcripts with the Plant Transcription Factor Database (PlnTFDB) by BLASTX, using default parameters and cut-off E-value of 1e^− 5^

Among all these TFs, a total of 131 TFs were found to be significantly differentially expressed at *P* ≤ 0.001, FDR ≤ 0.05, and log2|fold change| > 2 in relation to the color change; of these, 64 were upregulated and 67 were downregulated in stage II. In a previous report on saffron stigmas at anthesis, a total of 92 TFs were found to be upregulated in this tissue compared with their expression in leaves, corm, petals and stamens [21]. A list of the most up- and downregulated TFs in yellow samples compared to the white stage are presented in Fig. 4 and in Additional file 2: Tables S2 and S3, respectively.

The expression of a homolog to AT2G42680.1 (ATMBF1A), one of three genes in *A. thaliana* encoding multi-protein bridging factor 1, a highly conserved transcriptional coactivator, was increased by more than 5-fold. This was followed by a homolog of an unknown PHD finger family protein, encoding the gene AT2G18090, with an increase of over 4-fold; and by EIL (AT2G27050), which together with PIFs regulates growth in response to hormone and light signals [47]. In addition, other upregulated TFs were involved in circadian and light responses, such as PRR7 and PRR9 [48], which are partially redundant, are essential components of a temperature-sensitive circadian system; CCA1 and LHY, which have a positive effect on PRR7 expression levels [48]; and AT1G59940, which is rapidly induced by cytokinin, and is involved in red-light signaling, acting redundantly with ARR3 in the control of circadian period [49]. Previously, two TFs named CsULT1 and CsSAP09 were identified as regulators of apocarotenoid deposition in saffron [50, 51]. However, the expression patterns of both genes in mature flowers were not associated with apocarotenoid deposition or *CCD2* expression [8, 13], and neither CsULT1 or CsSAP09 were present among the upregulated TFs.

Among the downregulated genes, ASML2, which acts as a transcriptional activator of a set of sugar-inducible genes, was downregulated 6-fold [52], followed by a 5-fold downregulation of the bHLH TF AT1G09250, a negative regulator of cell elongation [53]; and AT1G06170, probably involved in the development of anthers.

### Identification of common, differentially expressed TFs among the Crocus species

The differentially expressed TFs identified in the saffron transcriptomes were investigated in the transcriptomes of the SI and SII stages of *C. cartwrightianus* and *C. ancyrensis*, in order to determine the presence of common TFs among the three species upregulated in the SII stages. Only 11 TFs that were previously identified as upregulated in the saffron SII transcriptome were found to be upregulated in *C. cartwrightianus* and *C. ancyrensis* SII stage transcriptomes. These TFs included homologs to ARF8, SPATULA, AT4G02720, Methylene Blue Sensitivity (MBS1), Homeobox-2 (HB-2), XRN3, Inducer of C-repeat binding factor (CBF) Expression 1 (ICE1), ALFIN, AT2G31410, AT5G28150 and Nuclear Factor YC (NF-YC). Auxin Response Factors (ARFs) contain a B3-DNA binding domain in the N-terminal region and mediate responses to auxin [54]. ARF8, together with ARF6, coordinates the transition from immature to mature, fertile flowers [55]. The overexpression of ARF5 from sweet potato significantly increased the content of carotenoids in transgenic Arabidopsis [56]. Previous studies demonstrated that in Arabidopsis, ARF5 affected lateral organ development, primary root initiation, cotyledon development and flower primordium initiation, while in tomato, ARF5 controlled fruit set and development [57]. A study on transcriptome profiling during the development of tomato fruits revealed a correlation between carotenoid content and ARF 4 and 6 expression levels [58]. In a similar study, an ARF TF was identified in orange-fleshed sweet potato [59]. The identified ARF in the present study showed 64 and 60% identity with tomato and Arabidopsis ARF8, respectively. Furthermore, in both plants, ARF8 has been reported to regulate fertilization and fruit development [60]. SPATULA (AT4G36930) is a bHLH (basic helix-loop-helix) TF involved in generating the septum, style and stigma in the female flower [61]. The TF encoded by AT4G02720 interacts with ARGONAUTE (AGO) RNA-binding protein, involved in RNA silencing [62] but also promotes the transcription of certain genes [63]. MBS1 (AT3G02790) has been shown to act downstream of β-cyclocitral, a β-carotene oxidation product, in singlet oxygen signaling [64]. In saffron stigmas, β-cyclocitral amounts increased from the earlier stages, reaching the highest level in the red stage, and decreasing afterwards [13]. β-cyclocitral also induces the expression of *CCD2* in saffron [20]. In addition, MBS1 interacts with the TFs MYB100 and MYB107, involved in flavonoid downregulation and suberin deposition, respectively [65] (Additional file 1: Figure S2). Another protein involved in retrograde signaling is XRN3, which plays a role in the processing and degradation of several classes of nuclear RNAs, including some involved in drought and high light stress [66], and acts as posttranscriptional gene silencing (PTGS) suppressors but also induces transcription [67]. Interestingly, XRN3 is also involved in the retrograde pathway between chloroplast and nucleus [68]. Homeobox-protein 2 (HB-2) is a regulator that processes light signals from phytochromes to alter morphogenesis during shade avoidance responses, and its overexpression causes long hypocotyls and reduced leaf expansion, as observed in etiolated seedlings [69]. In maize, rice and Arabidopsis*,* this TF is co-expressed with *PSY1* [70]. *ICE1* encodes a MYC-type bHLH transcription factor directly implicated in the major cold acclimation pathway [71]. The analyzed Crocus species begin their vegetative and reproductive growth coincident with an appreciable decrease of temperature, being able to tolerate temperatures below − 18 °C [72]. In addition, *CCD2* expression is induced by low temperatures, and low-temperature-responsive elements are present in its promotor [20]. Further, in potato tubers it has been shown that the reaction product resulting from carotenoid cleavage dioxygenase 4 (CCD4) activity has a role in temperature sensing in the plant [73]. Alfin is a family of proteins that bear an Alfin domain and a PHD domain at their N- and C-terminus, respectively, and bind to G-rich elements (GTGGNG or GNGGTG). The PHD domains are reported to bind to histone H3K4me3 peptide with varying methylation state preferences and binding affinities [74]. Among the histone H3 methyltransferases, SDG8 preferentially binds monomethylated H3K4 (H3K4me1), acting as an epigenetic regulator of carotenoid biosynthesis [75, 76]. The Alfin TFs are important regulators of plant development and are also involved in cellular phosphate homeostasis, a process in which other apocarotenoids, the strigolactones, are implicated [77]. NF-YC is a TF widespread in plants, animals, and other eukaryotes, and is also termed CCAAT Binding Factor (CBF) and Histone-Associated Protein5 (HAP5). The NF-Y complex consists of the subunits NF-YA, NF-YB, and NF-YC (Additional file 1: Figure S2), all of which are necessary for binding to the CCAAT box in the promoter regions of its target genes to regulate their expression [78]. NF-YC TFs function as important mediators of epigenetic marks in different plant developmental stages, implying an important function of these proteins in epigenetic regulation in plants. Arabidopsis NF-YCs mediate light-controlled hypocotyl elongation via modulation of histone acetylation [79]. One gene encoding geranylgeranyl diphosphate synthase 1 (GGPS1) is among the predicted functional partners of NF-YC (Additional file 1: Figure S2). Finally, AT2G31410 and AT5G28150 have no function defined, although the predicted functional partners of AT2G31410 include proteins involved in rRNA processing (Additional file 1: Figure S2).

### Expression analysis of the shared TFs in vegetative and reproductive tissues

**Fig. 5:**
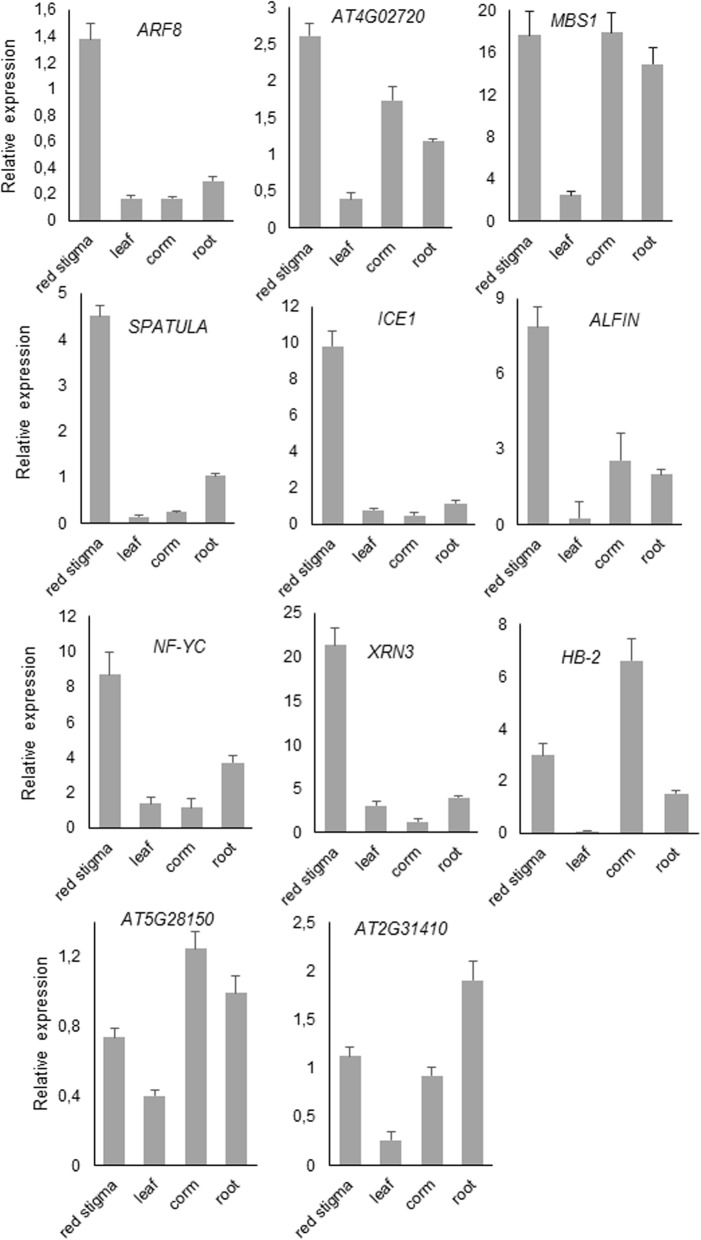
Relative expression levels of the 11 TFs by qRT-PCR in different tissues from saffron

The 11 upregulated TFs identified in the 3 Crocus species were selected for qRT-PCR analysis in different tissues of saffron, including the stigma tissue. The sequences of the primers used for qRT-PCR are listed in Additional file 2: Table S4. The expression levels of *ARF8*, *AT4G02720*, *SPATULA*, *ICE1*, *ALFIN*, *NF-YC*, and *XRN3* were higher in the red stigmas compared with the expression levels in roots, leaves and corm (Fig. 5). By contrast, *MBS1*, *HB-2* and *AT5G28150* showed higher expression levels in the corm, and *AT2G31410,* in the roots (Fig. 5). Of those contigs with higher expression levels in the stigma, *ARF8*, *SPATULA*, *ICE1*, *ALFIN*, *NF-YC*, and *XRN3* showed significant expression in the stigma compared with expression in the other tissues tested.

## Conclusions

In this study, we sequenced and assembled new transcriptome data from three Crocus species in two key stigma developmental stages for apocarotenoid biosynthesis and accumulation. The six transcriptomes obtained perfectly complement previous transcriptomes of saffron tissues [21, 22], providing new data of the earliest developmental stages in stigma development, when crocetin, crocin and picrocrocin biosynthesis begin [13]. A positive correlation among *DXS-CLA1*, *PDS*, *ZDS*, *Z-ISO*, *CrtISO*, *LYC-B*, *BCH-2*, *CCD2,* and *UGT74AD2* expression levels and apocarotenoid levels was observed in the three species. Therefore, the formation from phytoene to crocetin, and from crocetin to crocins appears to be principally under transcriptional regulation [3]. In emphasizing transcription factors, we compared the data from six transcriptomes in order to identify candidate regulators impacting apocarotenoid accumulation. Information on the spatiotemporal pattern of gene expression/metabolite accumulation can facilitate the understanding of gene function and the generation of a hypothesis for apocarotenoid biosynthesis control at the transcriptional level. We have identified 11 TFs that are expressed in the stigma tissue and correlated with apocarotenoid levels in the three species under study. These TFs belong to the ARF, bHLH, C2H2, HB, CBF/DREB1, ALFIN, and NF-YC families. Seven of them showed gene expression patterns that suggested a direct participation in the regulatory network influencing apocarotenoid profiles and content, and are involved in a complex network influenced by environmental factors and developmental stages known to regulate carotenoid accumulation in plants. Among these TFs, we found TFs involved in flower and fruit development, retrograde signaling, epigenetic modifications, and light and cold responses. The link between flower or fruit development and carotenoid accumulation in different plant species is associated with the process of chromoplast biogenesis [80, 81]. Chromoplast development in saffron is accompanied by massive synthesis and accumulation of apocarotenoids and changes in plastid ultrastructure [39]. Environmental changes also have an important impact on carotenoid biosynthesis and accumulation. The accumulation of apocarotenoids and flower development in saffron are associated with cold [3, 20], and both autumn and spring Crocus species produce and accumulate crocins during autumn-winter conditions in the Mediterranean area [25, 72]. In addition, in saffron and other Crocus species, synthesis and accumulation of crocins occurs in dark conditions [20, 82], as does the accumulation of β-carotene in carrot [83], and lycopene in some cultivars of red grapefruits kept in the dark [84], confirming how light modulates a complex network of signals connecting carotenoid biosynthesis, storage, and plastid development [85]. In addition, in this study TFs involved in retrograde signaling and epigenetic development have been identified. The network of signals generated by the plastid as it develops is known as retrograde signaling, and certain apocarotenoids act as signals in plastid-to-nucleus signaling [86]. However, not much is known about this network during the biogenesis of chromoplasts. By contrast, the role of epigenetic mechanisms in the control of carotenoid regulation is now well stablished [16, 87], therefore similar modes of regulation of apocarotenogenesis should also be present in plants.

## Methods

### Plant materials

Stigmas collected at different developmental stages were obtained from *C. sativus*, *C. ancyrensis* and *C. cartwrightianus;* also, leaves, roots and cormlets were collected from *C. sativus* grown under field conditions in the Botanical Garden of Castilla-La Mancha (Albacete, Spain). The tissues were dissected and frozen in liquid nitrogen and stored at − 80 °C until required.

### Extraction and analysis of crocins by UPLC-ESI-MS

Dissected samples from stigmas at two developmental stages were ground in liquid nitrogen with a mixer mill MM400 (Retsch GmbH, Haan, Germany), extracted with 30% MeOH and incubated for 10 min on ice. After centrifugation at 3000 g for 5 min at 4 °C, the supernatants were saved and stored at − 80 °C until further analysis by UPLC-ESI-MS. The UHPLC method used for the analysis and detection of crocetin, crocins and picrocrocin has been previously described [25]. In brief, a UPLC-PDA-qTOF-MS instrument (Waters, Spain) was used for compound separation and identification. Chromatographic separation was carried out on an Acquity BEH C18 column (150 × 2.1 mm i.d., 1.7 μm), with mobile phases consisting of formic acid∶water (1∶1000, *v*/v; phase A) and formic acid∶acetonitrile (1∶1000, v/v; phase B), in a gradient method of 80% A for 5 min, 80 to 25% A in 15 min, 25 to 0% A in 1 min, hold at 100% B for 9 min, and equilibration with 80% A for 4 min. The injection volume was 5 μL and the flow rate was 0.4 mL/min. Column and sample temperatures were 40 °C and 10 °C, respectively. UV-VIS spectra were acquired in the λ range 210–600 nm. The MS parameters were as follows: cone voltage, 30 V; capillary voltage, 3000 V; source temperature, 120 °C; desolvation temperature, 300 °C; cone gas flow, 50 L/h; desolvation gas flow, 500 L/h. MS data were acquired in centroid mode with a m/*z* range of 100–2000 with a scan and interscan time of 0.32 s and 0.1 s, respectively. MassLynx version 4.1 and Q-tof Micro version 4.1 (Waters, Spain) were used to control the instruments and calculate accurate masses. All the assays were performed in triplicate.

### Sample RNA extraction for sequencing

The RNA was extracted from stigmas at white (SI) and yellow (SII) stages using the Direct-zol Kit (Zymo Research, Freiburg, Germany). The obtained ARNs were quantified and between 10 to 15 μg from white and yellow stigmas were sent to Macrogen Inc. (Seoul, South Korea; www.macrogen.com) to be used for the construction of the libraries and further library sequencing. TruSeq RNA Sample Prep kit (Illumina, San Diego, USA) was used for the construction of the libraries corresponding to the three species and obtained from two different developmental stages of the stigmas. A HiSeq 2000 sequencer (Illumina) was used to generate inward paired-end reads of 100 bp.

### Data processing, transcriptome assembly and annotation

Adaptors and sequencing artifacts as well as low quality fragments were trimmed from all the obtained RNA-seq data using the NGS QC Toolkit. The high-quality reads were in silico normalized prior to de novo assembly in order to reduce the sequencing coverage of regions highly represented with a fragment density > 30×. Using this approach, the computational complexity was simplified keeping the quality of the assembled transcriptomes. The obtained sequences were assembled selecting a minimum fragment overlap of 40 bp, by using the de novo transcriptome assembler tool kit from Trinity [88]. Only those unigenes > 300 bp were included in the assembled transcriptomes and used for differential expression analyses, and gene ontology (GO) sequence annotation using Blast2Go [89].

### Analysis of differentially expressed transcripts

Employing the assembled six transcriptomes as a reference for *C. sativus*, the annotated libraries were subjected to transcriptome expression analysis. The six obtained libraries were analyzed using Bowtie (http://bowtie-bio.sourceforge.net/) [90]. The relative abundances of the aligned reads were estimated by using Cufflinks v.2.1.1 (http://cole-trapnell-lab.github.io/cufflinks/). Next, RNA-seq fragment counts were calculated by the unit of fragments per kilobase of exon per million fragments mapped (FPKM). A gene was considered expressed at low levels when for FPKM ≤2, moderately expressed for FPKM > 2 and ≤ 10, and highly expressed for FPKM > 10. For differential expression analysis, the values of log2 (FPKM+ 1) were calculated, and these were normalized by quantile normalization. The *P*-values were acquired by t-test between each sample, and fold changes were computed with the mean log2 (FPKM+ 1) values, gene by gene. R 2.14.1 (http://www.r-project.org) was used for differential expression analyses [91]. Differential expression between the two stigma stages was screened by detecting genes with statistical significance. In addition, the count matrix obtained for the sequenced samples was used to calculate a Euclidian distance matrix, which was used for hierarchical sample clustering. To identify the transcription factors (TFs), the assembled reads were searched against known TFs, as grouped in Plant Transcription Factor Database (PlnTFDB), using default parameters and a cut-off E-value of 1e^− 5^. PlnTFDB is an integrative library of plant TFs which offers complete lists of TF families from plants with fully sequenced genomes. The identified candidate transcription factors were validated in PlantTFcat (http://plantgrn.noble.org/PlantTFcat/).

### qRT-PCR analysis

For qRT-PCR analysis, total RNA (2 μg) from stigmas (red), leaves, roots and corms, was treated with RQ1 DNase (Promega, Madisson, USA) and reverse-transcribed using First-Strand cDNA Synthesis Kit (GE Healthcare Life Sciences, Buckinghamshire, UK) according to manufacturer’s instructions. The synthesized cDNAs were used for quantitative real-time PCR in a StepOne™ Thermal Cycler (Applied Biosystems, Foster City, California, USA). All reactions were performed in triplicate containing 10 ng of cDNA, LightCycler 480 SYBR Green I Master (Roche), and 0.2 μM specific primers (Additional file 2: Table S4) in a final volume of 10 μl. The results were analysed using StepOne software v2.0 (Applied Biosystems, Foster City, California, USA). The relative expression levels of the analysed target genes were normalized to that of the reference gene18 S rRNA as described previously [9].

## Additional files

Additional file 1: **Figure S1.** FPKM values distribution in the six analysed transcriptomes. **Figure S2.** Molecular interaction network of the 11 TFs identified as upregulated in all the Crocus transcriptomes in SII. Nodes represent proteins. The colored nodes are query proteins and first shell of interactors, and those which are filled is because its 3D structure is known or predicted. Interactions were produced using http://string-db.org. (PDF 643 kb)

Additional file 2: **Table S1.** TFs identified in the transcriptome analysis of stages I and II of *Crocus sativus*. **Table S2.** Up-regulated TFs in the SII/SI developmental stages of saffron. **Table S3.** Down-regulated TFs in the SII/SI developmental stages of saffron. **Table S4.** Oligonucleotide sequences used for qRT-PCR. (PDF 134 kb)

## Abbreviations

ALDH: aldehyde dehydrogenase
BCH: (β-carotene hydroxylase
CCD2: carotenoid cleavage dioxygenase 2
CDP-ME: 4-diphosphocytidyl-2C-methyl-d-erythritol
CDP-MEP: 4-diphosphocytidyl-2C-methyl-d-erythritol 2-phosphate
CrtISO: carotene isomerase
DMAPP: dimethylallyl pyrophosphate
DXP: 1-deoxy-d-xylulose-5 phosphate
DXR: 1-deoxy-d-xylulose 5-phosphate reductoisomerase
DXS: 1-deoxy-d-xylulose-5-phosphate
GAP: d-glyceraldehyde 3-phosphate
HDR: 1-hydroxy-2-methyl-2-(E)-butenyl 4-diphosphate reductase
HDS: 1-hydroxy-2-methyl-2-(E)-butenyl 4-diphosphate synthase
HMBPP: 1-hydroxy-2-methyl-2-(E)-butenyl 4-diphosphate
IDI: isopentenyl diphosphate isomerase
IPP: isopentenyl diphosphate
LCYB: lycopene-β-cyclase
MCT: 2C-methyl-d-erythritol 4-phosphate cytidyl transferase
MDS: 2C-methyl-d-erythritol 2,4-cyclodiphosphate synthase
ME-cPP: 2C-methyl-d-erythritol 2,4-cyclodiphosphate
MEP: 2C-methyl-d-erythritol 4 phosphate
PDS: phytoene desaturase
PDS: phytoene synthase
PSY: phytoene synthase
Pyr: pyruvate
UGT74AD2: *Crocus sativus* glucosyltransferase 2
ZDS: Z-carotene desaturase
Z-ISO: 15-cis-ζ-carotene isomerase
