# Supplementary material for: Multi-species transcriptome analyses for the regulation of crocins biosynthesis in Crocus
Source: BMC Genomics. 2019 Apr 27;20:320. doi: 10.1186/s12864-019-5666-5 (PMC6486981; doi:10.1186/s12864-019-5666-5)
Supplement: Supplementary file 1 — Figure S1. FPKM values distribution in the six analysed transcriptomes. Figure S2. Molecular interaction network of the 11 TFs identified as upregulated in all the Crocus transcriptomes in SII. Nodes represent proteins. The colored nodes are query proteins and first shell of interactors, and those which are filled is because its 3D structure is known or predicted. Interactions were produced using http://string-db.org. (PDF 643 kb) [file 12864_2019_5666_MOESM1_ESM.pdf]

Supplemental Figure S1

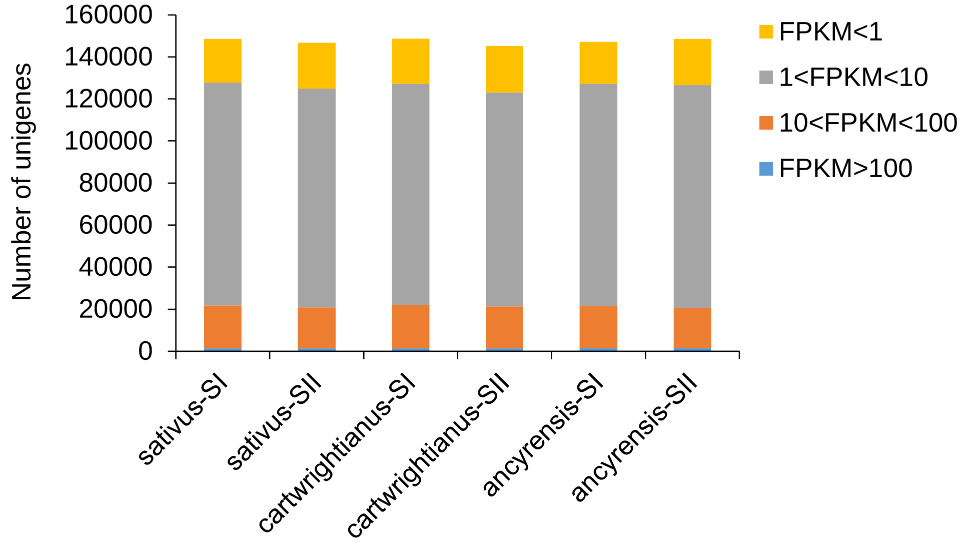

Supplemental Figue S1. FPKM values distribution in the six analysed transcriptomes.

Supplemental Figure S2

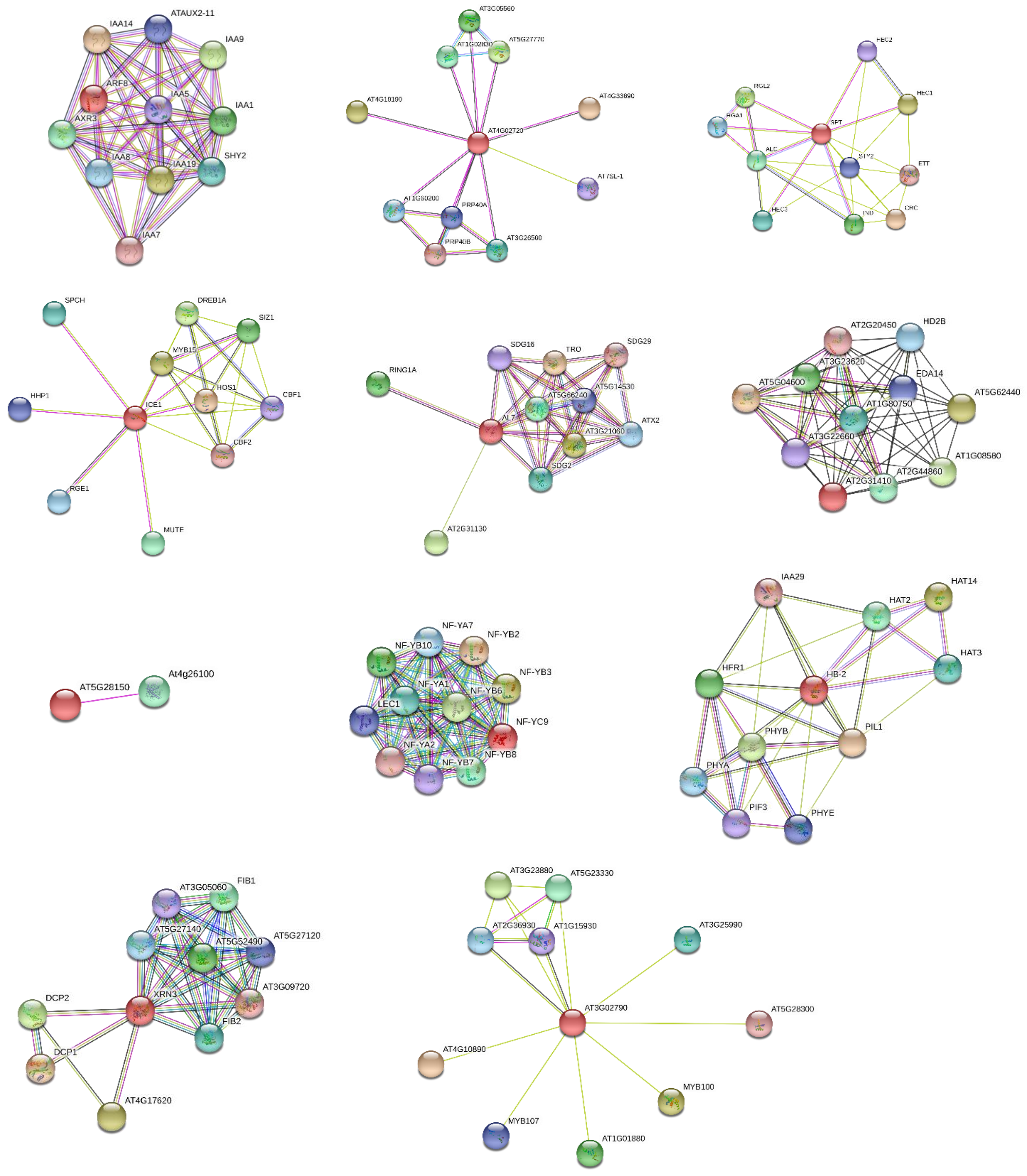

Supplemental Figure S2. Molecular interaction network of the 11 TFs identified as upregulated in all the Crocus transcriptomes in SII. Nodes represent proteins. The colored nodes are query proteins and first shell of interactors, and those which are filled is because its 3D structure is known or predicted. Interactions were produced using http://string-db.org.
