# Supplementary material for: Multi-species transcriptome analyses for the regulation of crocins biosynthesis in Crocus
Source: BMC Genomics. 2019 Apr 27;20:320. doi: 10.1186/s12864-019-5666-5 (PMC6486981; doi:10.1186/s12864-019-5666-5)
Supplement: Supplementary file 2 — Table S1. TFs identified in the transcriptome analysis of stages I and II of Crocus sativus. Table S2. Up-regulated TFs in the SII/SI developmental stages of saffron. Table S3. Down-regulated TFs in the SII/SI developmental stages of saffron. Table S4. Oligonucleotide sequences used for qRT-PCR. (PDF 134 kb) [file 12864_2019_5666_MOESM2_ESM.pdf]

Supplemnetal Table S1. TFs identified in the transcriptome analysis of stages I and II of *Crocus sativus*.

TF family	Number		% of all TFs	
	Stage I	Stage II	Stage I	Stage II
bHLH	53	39	8.98	6.32
Sigma70-like	1	1	0.17	0.16
NOT2/NOT3/NOT5	3	3	0.51	0.48
BBR-BPC	3	1	0.51	0.16
BES1	2	1	0.34	0.16
STAT	1	1	0.17	0.16
SNF2	1	0	0.17	0
mTERF	1	1	0.17	0.16
LSD	0	1	0	0.16
LHC	1	1	0.17	0.16
VOZ	1	1	0.17	0.16
TAZ	2	3	0.34	0.48
TBP	1	3	0.17	0.48
Trihelix	15	17	2.54	2.75
NF-YC	6	5	1.01	0.81
NF-YA	1	0	0.17	0
NF-YB	5	4	0.85	0.65
CO-like	7	6	1.18	0.97
DBB	4	5	0.68	0.81
AP2	5	5	0.85	0.81
NIN-like	1	1	0.17	0.16
HSF	8	9	1.35	1.46
B3	16	17	2.71	2.75
MIKC_MADS	6	3	1.01	0.48
TALE	3	3	0.51	0.48
Nin-like	4	3	0.68	0.48
ERF	13	15	2.20	2.43
E2F	5	5	0.85	0.81
AP2-EREBP	1	1	0.17	0.16
AP2/ERF-like	1	2	0.17	0.32
HD-ZIP	21	19	3.56	3.08
bZIP	20	22	3.39	3.56
BELL	1	1	0.17	0.16
LIM	1	1	0.17	0.16
NAC	9	18	1.52	2.91
YABBY	4	3	0.68	0.48
HB-PHD	2	2	0.34	0.32
TFB4	0	1	0	0.16
WD-40	3	2	0.51	0.32
WRKY	12	14	2.03	2.27
CAMTA	4	3	0.68	0.48
MYB	29	36	4.91	5.85
MYB-like	21	15	3.56	2.43
Jumonji	8	8	1.35	1.29
LBD	1	1	0.17	0.16
AHL	1	1	0.17	0.16
DDT	1	1	0.17	0.16
GRAS	18	17	3.05	2.75
SBP	10	8	1.69	1.29
ZF-HD	4	3	0.68	0.48
C2H2	19	19	3.22	3.08
G2-LIKE	12	9	2.03	1.46

MADS	2	3	0.34	0.48
ARR-B	7	6	1.18	0.97
TUB	5	4	0.85	0.65
DOF	0	3	0	0.48
SAP	1	0	0.17	0
EIL	2	2	0.34	0.32
C3H	12	10	2.03	1.62
HB-other	4	3	0.68	0.48
ARID	2	3	0.34	0.48
NF-YA	1	1	0.17	0.16
GeBP	5	5	0.85	0.81
HB-like	5	7	0.85	1.13
TCP	8	8	1.35	1.29
ARF	20	22	3.39	3.56
NF-X1	1	1	0.17	0.16
GATA-type	10	8	1.69	1.29
BES	4	4	0.68	0.65
LFY	1	1	0.17	0.16
SET	1	1	0.17	0.16
BET	2	2	0.34	0.32
bZIP	11	9	1.86	1.46
C2H2	9	9	1.52	1.46
M-type_MADS	0	1	0.17	0.16
TRF-like	2	2	0.34	0.32
TFIIS	16	19	2.71	3.08
FAR1	2	2	0.34	0.32
TRAF	2	1	0.34	0.16
OFP	2	3	0.34	0.48
PHD	24	22	4.06	3.56
VPS72/YL1	1	1	0.17	0.16
HMG	6	5	1.01	0.81
MBF1	3	3	0.51	0.48
LUG	1	1	0.17	0.16
WOX	1	2	0.17	0.32
EIL	1	1	0.17	0.16
S1Fa-like	1	0	0.17	0
CPP	3	3	0.51	0.48
PAS/LOV	1	2	0.17	0.32
TLP	1	1	0.17	0.16
CPP	2	2	0.34	0.32
FHA	1	0	0.17	0
SRS	2	0	0.34	0
BAH	1	1	0.17	0.16
kelp	0	1	0	0.16
G2-LIKE	1	0	0.17	0
GNAT	1	1	0.17	0.16
DMC1	1	1	0.17	0.16
SWI/SNF-BAF60b	2	2	0.34	0.32
Alfin-like	4	3	0.68	0.48
ORPHANS	Snl3<br>Srd2, ARR3,<br>PTAC6<br>GIF2<br>RR3<br>ELF3<br>PRR9<br>AT3G56220<br>AT3G47610	Snl3<br>Gif2<br>ELF3<br>PLL1<br>HOS1<br>AT3G56220<br>AT3G47610<br>AT3G53570<br>AT3G33520	2.88	2.27

	AT3G53570<br>AT3G12890<br>AT4G28590<br>AT4G21430<br>AT5G15020<br>AT5G28640<br>At1g61040<br>17	AT4G27310<br>AT4G21430<br>AT5G15020<br>AT5G28640<br>At1g61040<br>14		

Supplemental Table S2. Up-regulated TFs in the SII/SI developmental stages of saffron.

Contig name		TF family	Contig name		TF family
c2209	AT2G42680	MBF1	c32117	AT4G00990	Jumonji
c28898	AT2G18090	PHD	c2125	AT1G72360	AP2-EREBP
c12938	AT2G27050	EIL	c10494	AT3G26744	bHLH
c17461	AT5G57660	C2C2-Dof	c10539	AT4G38960	DBB
c1428	AT2G17390	bZIP	c65011	AT1G70070	NAC
c57354	AT5G65410	ZF-HD	c26700	AT4G37850	bHLH
c49033	AT1G33420	PHD	c26090	AT1G05805	bHLH
c47045	AT2G33880	WOX	c3998	AT5G55390	EDM
c1130	AT5G37020	ARF	c12070	AT3G62610	MYB
c3228	AT3G28910	MYB	c22229	AT5G14010	C2H2
c50239	AT1G63650	bHLH	c1394	AT3G02790	C2H2
c9930	AT3G61050	CalB	c26060	AT5G54630	C2H2
c11294	AT2G38090	MYB	c20120	AT1G58110	bZIP
c16158	AT4G02720	GRAS	c27634	AT4G34530	bHLH
c1425	AT4G26150	GATA	c27495	AT5G24330	PHD
c3228	AT1G08810	MYB	c23122	AT3G48430	C2H2
c4443	AT1G79350	PHD	c64463	AT1G03840	C2H2
c16303	AT5G26210	PHD	c11601	AT1G14350	MYB
c42753	AT3G17460	PHD	c3767	AT1G26960	HD-ZIP
c10956	AT2G47210	MYB	c13078	AT5G52660	MYB
c27271	AT1G05380	PHD	c11347	AT5G08430	SWI/SNF
c35259	AT1G75430	TALE	c62388	AT3G02990	HSF
c6341	AT1G03280	TFIIE	c9537	AT4G34590	bZIP
c8156	AT4G16110	ARR-B	c49728	AT3G13040	G2-like
c40764	AT1G66230	MYB	c68459	AT3G47610	TFIIE
c57354	AT5G65410	ZF-HD	c43909	AT2G01570	GRAS
c66895	AT1G59940	Orphans	c7427	AT3G12480	NF-YC
c72137	AT3G57670	C2H2	c51308	AT5G09410	CAMTA
c34317	AT1G13960	WRKY	c24041	AT5G02810	ARR-B
c21558	AT2G25640	SPOC	c6399	AT1G75660	Orphans
c1391	AT4G36930	bHLH	c20829	AT2G31410	Orphans
c69985	AT5G28150	bHLH	c30831	AT4G16780	HB

Supplemental Table S3. Down-regulated TFs in the SII/SI developmental stages of saffron.

Contig name		TF family	Contig name		TF family
c17352	AT1G63490	Jumonji	c26965	AT1G05055	TFIIE
c14264	AT1G01520	MYB	c12641	AT3G51060	C2H2
c2544	AT3G12890	Orphans	c16104	AT4G37650	GRAS
c26567	AT1G06170	bHLH	c24956	AT4G00260	B3
c42801	AT1G09250	bHLH	c35025	AT5G23405	HMG
c15659	AT2G40140	C3H	c1373	AT2G40970	MYB
c56080	AT3G25790	G2-like	c14117	AT2G31280	bHLH
c62716	AT3G09735	S1Fa-like	c18746	AT4G09960	MADS
c17001	AT5G05550	Trihelix	c1602	AT3G19360	C3H
c45384	AT5G05090	G2-like	c36623	AT2G44730	Myb/SANT
c33486	AT5G03150	C2H2	c47386	AT3G47640	bHLH
c54934	AT5G10970	C2H2	c22762	AT4G25210	GeBP
c43877	AT1G05690	G2-like	c36282	AT4G30935	WRKY
c56488	AT1G51190	AP2	c1598	AT2G22430	HD-ZIP
c60040	AT1G47870	E2F/DP	c25751	AT1G01060	MYB
c42365	AT4G28590	-	c3631	AT5G56860	GATA
c40378	AT3G53310	B3	c15558	AT1G18340	TFIIE
c62721	AT5G53210	bHLH	c13154	AT1G01160	-
c42285	AT5G58900	MYB-related	c21222	AT3G60530	GATA
c42817	AT5G41315	bHLH	c13010	AT5G29000	MYB
c23395	ZKSCAN1	C2H2	c64168	AT2G02710	AP2
c41738	AT4G04580	G2-like	c45569	AT3G13940	RRN3
c38166	AT5G18240	G2-like	c30555	AT3G21330	bHLH
c60348	AT3G59060	bHLH	c62615	AT5G57390	AP2
c53465	AT5G35770	SAP	c2452	AT3G58680	MBF1
c19773	AT1G27660	bHLH	c17791	AT1G09540	MYB
c31174	AT4G37180	MYB	c42839	AT5G42520	BBR/BPC
c47508	AT4G31680	B3	c18474	AT1G25540	-
c17664	AT2G22540	MADS	c27822	AT4G28910	TIFY
c55157	AT5G67450	C2H2	c2111	AT4G18020	ARR-B
c54593	AT1G10586	bHLH	c17889	AT1G45249	bZIP
c47923	AT5G25190	ERF/AP2	c10084	AT5G48560	bHLH

Supplemental Table S4. Oligonucleotide sequences used for qRT-PCR.

contig	homolog	Oligonucleotide 5'-3'	Oligonucleotide 3'-5'
c1130	ARF8	AGCGGTCGAGCTTTAGAGATCA	GAGAAATCCAGAGGCGGGAACA
c16178	AT4G02720	CGTGGAAATCGAAGGTGTGG	GTACCTGCCCGATATCAGCT
c45953	MBS1	CTGGTCAGTTAGCACAGGGT	CAGGGACCGTTCAGTGAGAT
c6399	XRN3	ATCTGCCTCCAGCCAGGATC	AGATCGACGGCGTCATGATC
c10494	ICE1	GCAATGAGCTTCGACGCCT	CGGCAGTGATAAGGGGAAG
c8036	ALFIN	CATTTCTCGCAGAGGTCAC	CTGGGAAGAGCAAAGTGAG
c69985	AT5G28150	CGTGGAAATCGAAGGTGTGG	GTACCTGCCCGATATCAGCT
c20829	AT2G31410	TCCTTCTCCTTGGTACGGAC	AGGTCGACGAAGCGGATC
c1391	SPATULA	CGAAACTTCATTTGGGATGG	CAGATGATGCTTGCCCTACA
c30831	HB-2	GTAATGAGCTGCGGGAAGAG	CGGTTCTGGAACCAGACTTC
c7427	NF-YC	GTCCTGTCACAAAGATCTTGCA	AATGAGGAAGAAGCTCGGCA
